# Forgotten Grievers: The Unheard Voices Of Grandparents Grieving For Grandchildren From The “Swords of Iron” War

**DOI:** 10.1093/geroni/igaf122.095

**Published:** 2025-12-31

**Authors:** Ile Kermel-Schiffman, Rinat Lifshitz, Ayala Eliyahou, Galia Segev-Rosenberg

**Affiliations:** Ariel University, Ariel, HaMerkaz, Israel; The Max Stern Academic College of Emek Yezreel, Emek Yezreel, HaZafon, Israel; Bar-Ilan University, Ramat Gan, HaMerkaz, Israel; Kfar Saba Municipality, Kfar Saba, HaMerkaz, Israel

## Abstract

Since the Swords of Iron War began on October 7, 2023, approximately 4,000 grandparents in Israel have lost a grandchild. Research shows their bereavement differs significantly from that of parents, spouses, or siblings, yet they often remain an overlooked group. This study examines (1) grandparents’ unique bereavement experiences and (2) their needs, vulnerabilities, and resilience in coping with grief. Using a qualitative approach, 25 grandparents who lost grandchildren in military service or terrorist attacks were interviewed. Data were analyzed through an inductive five-step qualitative content analysis. Three key themes emerged: (1) “Losing a grandchild is like nothing else”-participants described deep emotional, cognitive, physical, and behavioral distress; (2) “What is my place now?”- they struggled with identity shifts and sought formal recognition as bereaved family members; and (3) “We learned to live with the pain” - resilience and coping strategies shaped their adaptation. Bereaved grandparents require tailored support and formal acknowledgment, yet they also demonstrate resilience. Professional interventions - both individual and group-based - are crucial to addressing their grief while maintaining their family roles. This study broadens discourse on grandparents’ bereavement, providing a foundation for targeted policies and interventions to enhance their well-being.

